# Non-Destructive Investigation of Intrinsic Magnetic Field of Austenitic Biomaterials by Magnetic Field Sensors

**DOI:** 10.3390/s22239120

**Published:** 2022-11-24

**Authors:** Milan Smetana, Daniela Gombarska, Zuzana Psenakova, Vladimir Chudacik

**Affiliations:** Department of Electromagnetic and Biomedical Engineering, Faculty of Electrical Engineering and Information Technology, University of Zilina, Univerzitna 8215/1, 010 26 Zilina, Slovakia

**Keywords:** fluxgate sensor, intrinsic magnetic field, austenitic stainless steel, heat treatment method

## Abstract

Investigation of the intrinsic magnetic field of austenitic biomaterial specimens after various heat-treatment processes and mechanical deformation is a matter in this study. Both heat-treatment and mechanical deformation influences are under investigation. A new approach incorporates innovative solutions with the goal to increase the resolution of gained signals in contrast to conventional methods. The proposed procedure was tested on real material specimens. A magnetic field sensor (fluxgate type) was used for this purpose. The presented results clearly show that gained signals can be increased when the appropriate probe instrumentation is used, and the characteristics are further mathematically processed.

## 1. Introduction

The use and development of biomaterials are continuously among dynamically expanding industries. The reason is mainly humanity’s focus on quality and length of life, access to applicable therapeutic, and assistance functions of new materials, devices, etc. According to the statistics of various organizations such as WHO (World Health Organization), SAR (Slovak Arthroplasty Register), and EAR (European Arthroplasty Register), the number of implanted biomaterials (especially artificial joint replacements) has increased over the last decades. However, due to the implantation of components into the body and their increasingly frequent interaction with people, it is necessary to control these components made of biomaterials. The control must be carried out immediately before implantation into the biological environment, and of course, these biomaterials must also be continuously controlled during their use. This gives a wide scope for the implementation of non-destructive investigation using appropriate methods.

Biomaterial can be defined as any synthetic material that is intended to replace or restore the function of body tissue and is in continuous or occasional contact with body fluids [1].

A biomaterial is a non-living material used in medicine, intended for interaction with the biological environment. The use is very broad: they largely serve as a replacement for some parts of the skeleton and the locomotor system, and their use in the assistance function of the cardiovascular system (artificial heart valves) is also important. Equally important is the creation of an interface between medical devices and the human body, for example, during long-term monitoring of physiological functions or surgical interventions. Such use requires a wide spectrum of different types of biomaterials for different specific purposes. Metals, polymers, ceramics, glasses, or alloys are used as biomaterials in such cases [1,2,3].

An ideal biomaterial (or a combination of biomaterials) should meet the following properties:

- biocompatible chemical composition to prevent adverse interactions between biological tissue and biomaterial,
- it should have the necessary resistance to degradation (for example, resistance to biological degradation in the case of polymers, resistance to corrosion in the case of metals),
- acceptable strength to maintain regular and long-term loads (joints),
- high wear resistance to minimize the formation of friction,
- in specific cases, biodegradability and bioactivity are required.

The interaction of biomaterials and biological structure therefore places high demands on the quality, stability, durability, and biocompatibility of these materials [2]. Material inhomogeneities, which can arise in biomaterials, especially during use after implantation, are mainly of fatigue and corrosion nature. However, the fact that austenitic materials change the mechanical and electromagnetic properties due to mechanical stress when in use is also important. This is an important fact, as, based on theoretical knowledge and appropriate control, it is possible to prevent conditions that can ultimately be life-threatening for the patient.

The properties of all materials can be described by dividing them into mechanical and electromagnetic. Mechanical properties describe and define structural integrity in terms of resistance to the external environment and workability. Electromagnetic properties describe and define the force action of electric and magnetic fields of the environment on material and its action on other materials. In terms of magnetic properties, for example, the eddy current method is suitable for checking and investigating paramagnetic conductive materials. In this work, the subject of investigation is stainless corrosion-resistant steel (austenitic steel), whose relative permeability values belong to the area of paramagnetic materials and, in a special case, to ferromagnetic materials (for example, caused by the transformation of austenite to martensite during mechanical deformation) [4].

Corrosion-resistant or stainless steels are a type of material where the main required property is their corrosion resistance. The corrosion resistance of corrosion-resistant materials is primarily determined by the protective passivation layer created on their surface. This group includes alloy steels with a chemical composition that guarantees their high resistance to corrosion in the various environments. They contain a lot of rare alloying elements, and the method of their production is energy-intensive. The optimal choice of steels for demanding conditions in aggressive environments requires expert knowledge, and experimental verification in model conditions imitating workload is also appropriate. Stainless steels are a relatively large group and can be divided into the following groups: austenitic, martensitic, ferritic, duplex, and precipitation curable [5,6,7].

MF sensors have been applied for eddy currents testing usually in the inspection of covered or buried defects. In 2000, Avrin [8] proposed a new eddy-current system based on low-noise magneto-resistive (MR) sensors for cracks and corrosion detection in thick, multi-layer metal structures. It was able to detect narrow flaws in the lowest layer of a stack of three aluminium plates.

The technique for automatic detection in third-layer cracks at rivet sites in aircraft structures using the response signals collected by giant MR sensors (GMR) was developed by Yang et al. The system contained pulsed waveform as the excitation source of a multi-line coil and sensed the transient fields of the induced eddy currents using a GMR sensor [9].

Rosado et al. [10] used magneto-resistive sensor for the detection of surface-breaking defects using higher frequencies. They designed a MR sensor-based EC probe for the detection and characterization of surface-breaking defects. The designed probe and used techniques improved the signal to noise ratio at which defects can be detected and the relative variation of the measured signal when operating at high frequency.

Study [11] used magneto-resistive sensors for wire rope inspection in practical applications and discussed comparison with other methods of testing.

Plastic deformation is considered an irreversible change in the shape of the material (permanent deformation) due to the action of external forces, without the appearance of cracks. Cold work plastic deformation takes place below the recrystallization temperature point. It is caused by the movement of dislocations and results in irreversible shape changes. An accompanying phenomenon is the strengthening of the material. Mutual displacement of mass in plastic deformation processes occurs in two ways: diffusion and non-diffusion. The microphysical nature of the diffusive mass transfer is caused by the presence and movement of vacancies and interstices. During this deformation, rows of atoms rotate at a high speed by a certain angle concerning the direction of displacement. A doubling of the crystal occurs. Thus, the material properties of steel are significantly influenced by the processing method and production conditions. Depending on the quantity of ferromagnetic components, steels exhibit different magnetic properties, ranging from paramagnetic to ferromagnetic. Austenitic steels also show the presence of a magnetic field that was induced during production. One of the main factors influencing the residual magnetism is the heat treatment parameters. The residual magnetism of such steels can be partially removed by appropriate heat treatment.

An ECT probe with an array of tunnel magnetoresistance (TMR) sensors and two excitation coils was proposed by Wang et al. in [12] for the inspection of surface and subsurface faults. Various frequencies and amplitudes of alternating currents are concurrently driven through the two coils. The resistance versus magnetic field curves for TMR sensors are nonlinear. The high-frequency signals from the sensors must be calibrated using a non-linear calibration approach based on the non-linear feature.

Using a TMR sensor instead of a detecting coil, researchers in [13] created two different types of tiny ECT probes—single- and dual-channel tunnelling magnetoresistance (TMR) probes. The induction coil’s dual TMR sensor probe may produce a reliable crack signal without being impacted by magnetization fluctuation. A signal change with a high signal-to-noise ratio was visible at the crack point in the line-scanned differential signal intensity and phase. The crack’s length and width affect these signals [14].

Recent developments in fluxgate magnetic sensors were summarized by Wei et al. [15]. The primary tool for identifying weak magnetic fields is fluxgate sensors. They have so far been used in numerous industries, including non-destructive testing, wearable electronics, and geophysics and astronomical studies.

The manufacture and characterization of a flexible, flat, miniature fluxgate sensor with a thin, rectangular, amorphous magnetic core created using the pad/printing process is shown in Paper [16].

Fundamental research is being done on the performance of orthogonal fluxgate sensors with meander-shaped cores [17]. The meander-shaped cores are created using a micro-patterning technique based on a ribbon made of cobalt. The fundamental benefit of this arrangement is that, without disrupting the excitation process, the linear operating range of the sensor may be changed simply by altering the number of strips.

The case paper [18] presents a novel integrated microfluidic platform for magnetic bead detection and trapping based on micro-coils and micro-fluxgate. The magnetic beads are trapped in a micro-spiral coil created using microfabrication technology, and a micro-fluxgate is used to detect the weak magnetic field that is created as a result. The study’s findings show that the microfluidic system is a good choice for biomarker collection and detection since it efficiently captures and detects magnetic beads.

The micro-fluxgate sensors have demonstrated remarkable sensitivity for the detection of the magnetic beads in the study [19]. Co-based amorphous ribbons with high permeability and low saturation magnetic induction were used as the core materials for portable magnetic biological detection to reduce the operational requirements of fluxgate-based magnetic beads detection.

Smetana et al. used a fluxgate sensor to measure the intrinsic magnetic field after applying plastic deformation to various deformation levels of various austenitic steel kinds while studying them under the same conditions. The results obtained demonstrate the significant impact of deformation on austenitic biomaterials’ magnetic fields. The findings of this study are described in the publication [20,21].

In this paper, austenitic corrosion-resistant steels are mainly examined. These steels’ exact composition resulted in their austenitic structure. It is accomplished by striking a balance between components that make ferrite and austenite (Ni, Mn). Compared to ferritic corrosion-resistant steels, austenitic steels have a 30% lower thermal conductivity, a 50% higher thermal expansion, and better toughness and ductility. Their face-centred cubic lattice provides these characteristics.

This paper is organized as follows. The experimental setups for both performed experiment types are briefly described in Section 2. Section 3 is divided into two parts; Section 3.1 describes the experimental results for the investigation of heat treatment influence on the intrinsic magnetic field in examined specimens and Section 3.2 contains the experimental results of investigation of cold work plastic deformation on the intrinsic magnetic field of selected material specimens. Section 4 brings conclusions with a summary of the findings.

## 2. Experimental Setup

**Figure 1:**
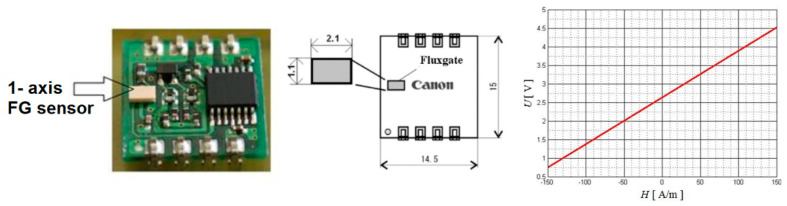
Fluxgate sensor used in measurements with its calibration curve.

The main goal of this work is evaluation of the intrinsic magnetic field of austenitic biomaterial specimens after previous mechanical deformation. The first series of measurements aim to point out the effect of heat treatment of AISI 316L austenitic steel on its intrinsic magnetic field. A commercial fluxgate-type sensor, Figure 1, was used for the measurement, along with other peripheral devices and virtual instrumentation. As a magnetic field sensor for a vector magnetic field has the fluxgate magnetometer typical range which allows for the measurement of the earth’s magnetic field, and its resolution is far below one ten thousandth. It has historically been used for prospecting, metal detecting, navigation, and compass work. Designs for fluxgate magnetometers can be generally divided into two categories: with rod cores and utilizing ring cores. Many other designs, mainly based on rod cores, exist, but none have attained the level of development and efficiency of the two abovementioned kinds [14,22,23].

For individual measurements, we used a single-axis commercial fluxgate sensor (Canon, Tokyo, Japan), which is suitable for measuring relatively weak magnetic fields. This sensor measures magnetic field values in the frequency range *f* = <0, 3.4> kHz. For our case, we used the measurement of the stationary (one-way) component in the so-called sensitive axis of the sensor. The orientation of the sensitive axis of the sensor with respect to the investigated material was such that the normal component of the magnetic field was recorded. The sensor, together with the electronic circuits, converts the value of the magnetic field intensity to the output DC voltage in the defined range according to the calibration curve, Figure 1.

For measurements, the software LabVIEW version 2015 (National Instruments) was used, in which an interface was created. 

**Figure 2:**
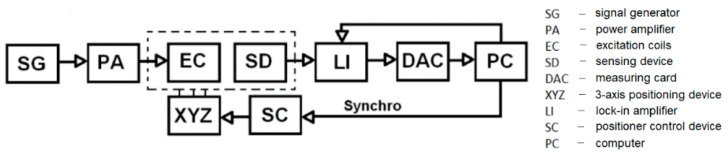
Block diagram of the individual components’ connection during the measurement.

The requirements for the interface are determined as follows: part of the actual measurement and recording of useful signals will be performed using LabVIEW and analysed using selected mathematical operations. Non-destructive investigation requires a few initial settings that must be implemented in the VI and made available on the user interface:

- setting the scanning parameters,
- defining the region of interest (ROI),
- defined positioning of the probe above the material and its movement in three axes. The diagram of the connection of individual devices is shown in Figure 2.

**Figure 3:**
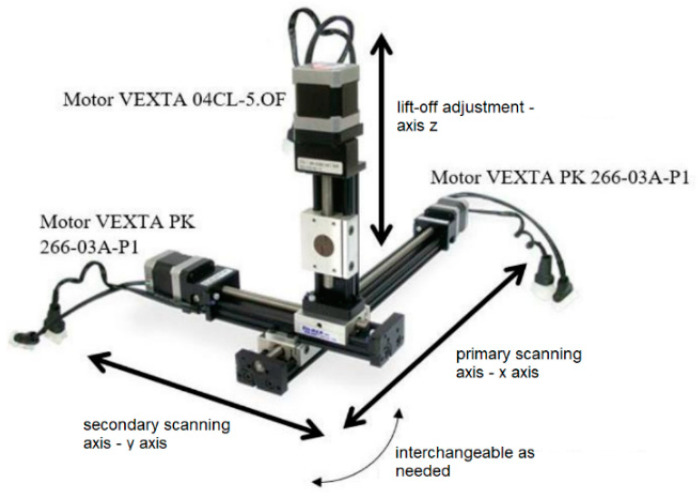
3-axis independent linear positioning device with step motors.

When performing the measurements, the main element of the measurement chain is a fluxgate-type sensor. A signal generator is used to ensure its power supply to its electronic part, while the power amplifier block is not used in this case. In this measurement, additional excitation of the EM field is not necessary, because the sensor detects the response of the magnetic field above the material under investigation. The measured output value of the sensor was read after filtering by a lock-in amplifier and stored in the computer. The entire process, controlling the position of individual motors on the platform on which the sensor is placed, was controlled by a PC. The used device for 3-D positioning is shown in Figure 3.

The area of interest on the surface of the biomaterial being examined is always determined by the length of the scanned row/column and the step between the rows/columns. The entered distance in millimetres is converted to the number of motor steps. The probe scans a square or rectangular area depending on settings. Another important part is setting the initial lift of the probe above the material. Considering the principle of the used electromagnetic investigation methods, it is obvious that with the increase in the distance of the measuring probe from the investigated surface, the information value of the resulting signals decreases. The lift-off parameter that defines this setting is determined by a value entered by the user, with the probe touching the surface of the material before the start of scanning. The setting of the scanned area is given by three inputs: line length, line width, and number of lines. The interface also works similarly in the case of scanning columns.

### 2.1. Investigation of the Heat Treatment Influence

Specimens were prepared from AISI 316L steel. The biomaterial in question is paramagnetic in its initial state. However, after mechanical processing (e.g., cold drawing), the magnetic properties change due to dislocation movement and twinning deformation. The consequence is the formation of an induced martensitic structure, which is partially ferromagnetic. This can cause a memory effect inside the material. Such a structure also contains a small percentage of the so-called δ-ferrite, which exhibits ferromagnetic properties. Stabilization of the properties of such material is achieved using the heat treatment mechanism. At temperatures up to *T* = 400 °C, regeneration of martensite deformation occurs, which transforms back to austenite. When the temperature *T* = 850 °C is reached, a recrystallization process was observed, during which the number of dislocations in the material reduced. At temperatures above *T* = 1150 °C, the chemical composition of steels is regularized, while inclusions dissolve. Moreover, at this temperature, δ-ferrite may be removed from the structure.

**Figure 4:**
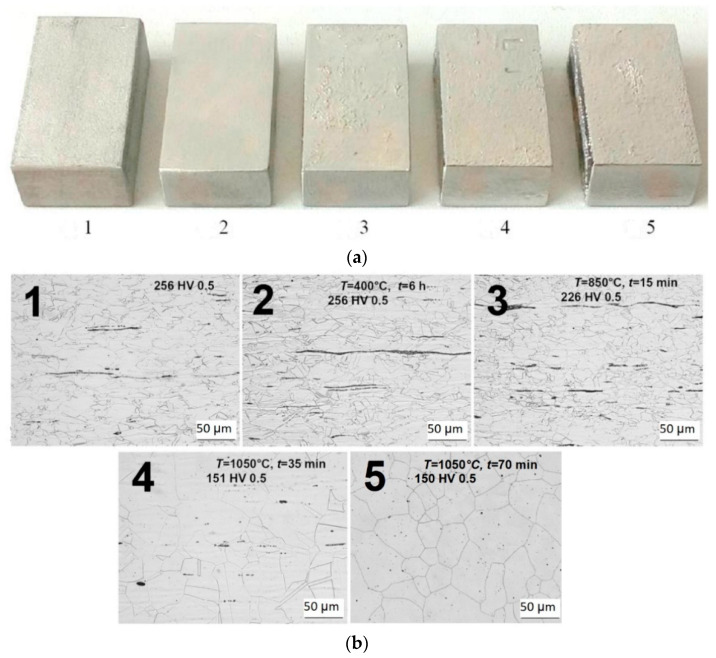
Investigated specimens of biomaterial AISI 316L. (**a**) Material specimens (**b**). A microstructure of specimens.

The examined specimens have a defined cuboid shape with length *l* = 30 mm, width *w* = 20 mm, and height *h* = 10 mm, Figure 4a. Individual specimens differ in heat treatment conditions. The heat treatment parameters are listed in Table 1.

For visual verification of the structure of the heat-treated material, a scan of the specimen structure was performed using a scanning electron microscope, where the result is shown as a microstructure in Figure 4b. The microstructure of the investigated material after the surface polishing process is shown with 500 times magnification. A solution consisting of HF, HNO_3_, and glycerine was used for polishing.

**Figure 5:**
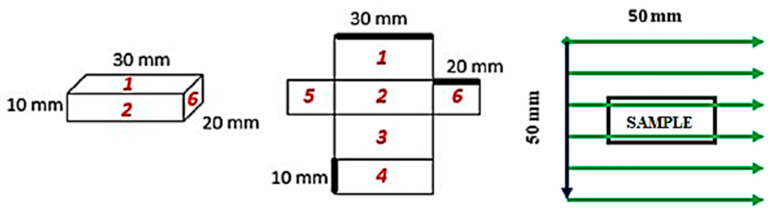
Biomaterial specimen dimensions, numbering, and distribution of scanning points for heat treatment measurements.

The subject of the investigation are all sides of the material specimens, which are successively examined according to the numerical order shown in Figure 5. Area scanning of the sides of the specimen is carried out on an area *s* = 50 mm × 50 mm, while the sensor is placed above the surface of the specimen at a height of *h*_s_ = 1 mm. 100 horizontal lines were scanned with a spacing of *y* = 0.5 mm with the number of measured samples *n*_s_ = 1000 at a sampling frequency of *f*_s_ = 100 Hz. The material specimen was placed in the centre of the scanning area and the scan started from surface 1, Figure 5.

### 2.2. Investigation of the Mechanical Deformation Influence

The next part of the research deals with the investigation of the influence of changes in the electromagnetic properties of conductive biomaterials because of mechanical deformation on the investigated biomaterial. As a result of external force, under certain circumstances, plastic deformation (PD) occurs, which is an irreversible event associated with a change in the properties of the material. In the case of austenitic corrosion-resistant steels, this manifests itself, in addition to the change in shape and mechanical properties, mainly in the change of electromagnetic parameters, which is a very important fact. A typical consequence of the action of external forces on the investigated biomaterial is a local change in its microstructure. According to theoretical knowledge, austenitic structures are transformed into martensitic ones, the so-called phase transition. This transformation results in a local weakening of the biomaterial, which is thus more susceptible to the formation of corrosion and fatigue inhomogeneities [24,25]. Due to the significant change in the material magnetic properties, suitable sensors should be used to detect changes in the magnetic field and the transition in the properties of the biomaterial in a non-destructive way. We used a single-axis commercial fluxgate sensor utilized likewise in the previous experiment.

The investigated specimens are made of austenitic steels AISI 304, AISI 316L, and AISI 316Ti. The manufactured specimens are made from a single piece (bar) of cold-rolled steel in a cylindrical shape. After cutting to defined dimensions, the specimens were annealed due to the elimination of the magnetic field after cutting, as mechanical stress is on the material. 

The situation during the preparation of the specimens was as follows: at the beginning, five material specimens with the same dimensions and starting properties were available. All specimens were annealed before deformation at a temperature of *T* = 850 °C for a period of *t* = 15 min. The radius *r* = 6 mm and the height *h* = 15 mm were the reference values of the dimensions. One of the specimens from each type of steel was designated as reference (VS), on which the process of mechanical deformation did not occur.

**Figure 6:**
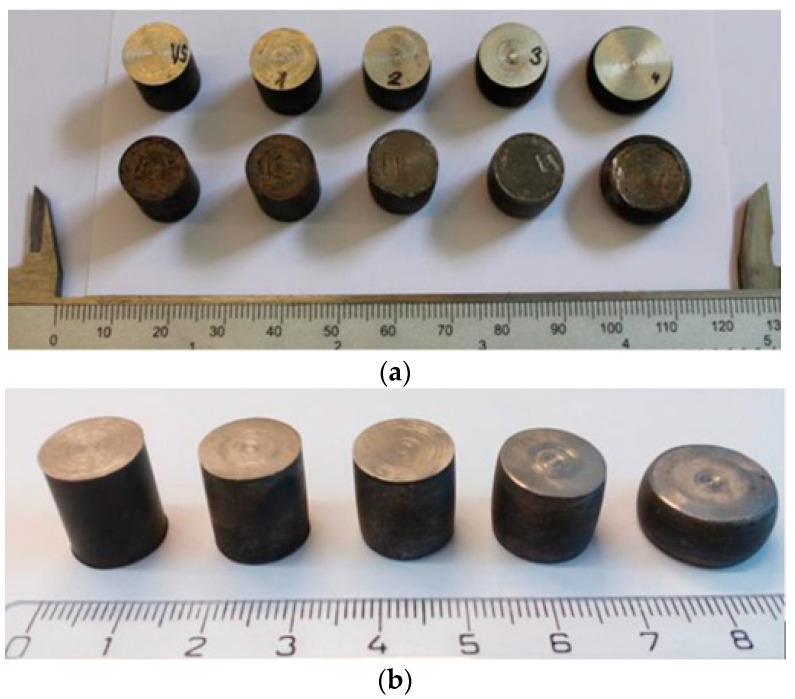
Investigated specimens of AISI 316L austenitic steel (**a**) with a mechanically-treated surface and (**b**) without additional mechanical surface treatment, all stages of plastic deformation.

Figure 6 shows an undeformed specimen (first from the left) and four specimens with gradual deformation. The picture shows in part (a) set of the specimens with a mechanically-treated surface after annealing and part (b) set of the specimens selected for an investigation that did not undergo additional mechanical surface treatment. Several series of defined manufactured specimens were investigated to rule out a processing error, or specimen preparation, which would lead to irrelevant measurement results.

**Figure 7:**
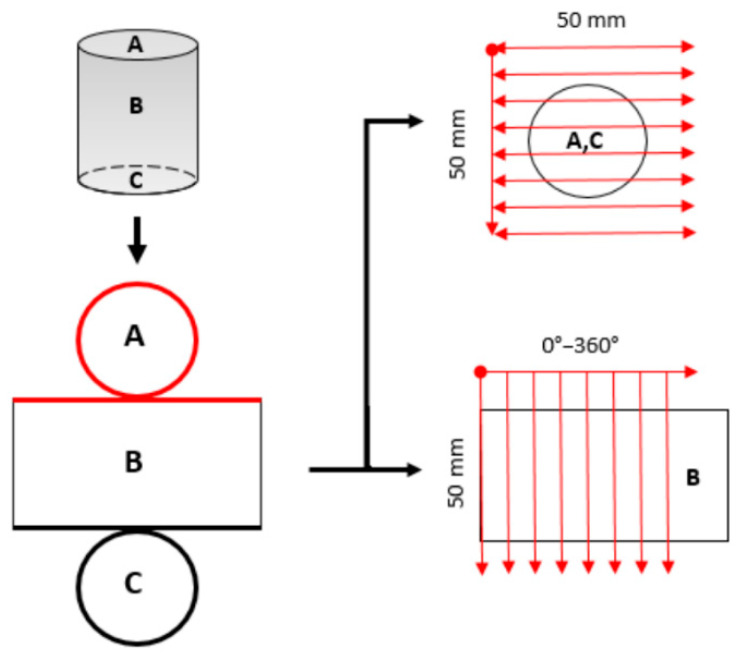
Procedure for scanning individual surfaces of biomaterial specimens for deformation influence experiment.

The measurement was carried out in three steps, which were identical for all specimens. First, the upper platform was scanned, then the lower platform, and finally the entire circumference of the specimen shell was scanned. All parts and the specimens were scanned separately. Surface scanning of the platforms was carried out on an area *S* = 50 mm × 50 mm, while the sensor was placed above the surface of the specimen at a height of *h_lo_* = 1 mm. *n_r_* = 100 horizontal lines with a space of *y* = 0.5 mm are scanned with the number of samples *n*_s_ = 1000 at a sampling frequency *f_s_* = 100 Hz. In this way, a two-dimensional comb scan of the area of interest was created, while the examined specimen was placed in the centre of the scanned area. When scanning the shell of the specimen (quasi-cylindrical shape due to mechanical deformation), *n_r_* = 16 lines with a length of 50 mm with an angular spacing of *φ* = 22.5° were measured. The specimen was placed in the middle of the scanned area. An illustrative display of the layout is in Figure 7. The data taken from the shell of the specimen are numerically refined with additional calculated samples from other angles, as it was not possible to make a finer network of measured samples.

## 3. Results 

Two types of experiments were performed: heat treatment influence on the intrinsic magnetic field of the specimen and plastic deformation influence on the intrinsic field. Measurement setups are described in the previous part of the paper.

### 3.1. Heat Treatment Investigation Results

**Figure 8:**
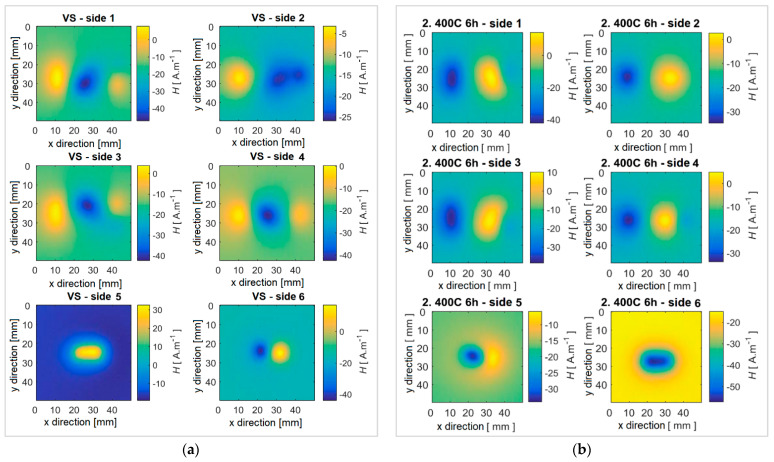
Distribution of the magnetic field of the specimen (**a**) No. 1 and (**b**) No. 2.

**Figure 9:**
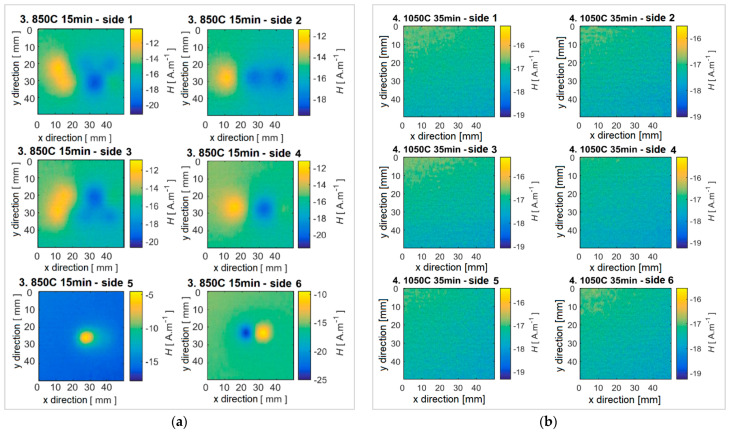
Distribution of the magnetic field of the specimen (**a**) No. 3 and (**b**) No. 4.

Experimental results achieved using the fluxgate sensor on specimens with different heat treatments are presented. The obtained data were processed and plotted for better clarity as the surface distribution of the magnetic field on the surface of the specimen. The reference specimen No. 1 without heat treatment shows the strongest signal response, and thus the highest density of ferromagnetic components, Figure 8a. For specimens with a gradually increasing annealing temperature, a decrease in the detectable intensity of the magnetic field occurs. Specifically, with specimens No. 2 and No. 3, the field distribution is still detectable by the sensor, Figure 8b and Figure 9a. However, when applying solution annealing, specimens No. 4 and No. 5, the intrinsic magnetic field it is not measurable, Figure 9b. The reason may be insufficient sensitivity of the fluxgate sensor or the disappearance of ferromagnetic components in the examined material. Based on the images from the microstructural analysis, it can be assumed that the second statement is valid. It can therefore be concluded that by suitable heat treatment, the ferromagnetic components are partially or completely removed, which is reflected in a clear decrease in the intensity of the specimens’ intrinsic magnetic field. In all investigated specimens that show a measurable presence of a magnetic field, it shows the presence of two or three local maxima. The examined specimens thus had magnetic components distributed evenly, which means that these were a consequence of the production process, and were not induced additionally, for example, by mechanical intervention. As can be seen in specimen 3, these components are distributed evenly on the surface, and it was not noted that the influence and procedure of heat treatment affected any part of the specimen earlier.

**Figure 10:**
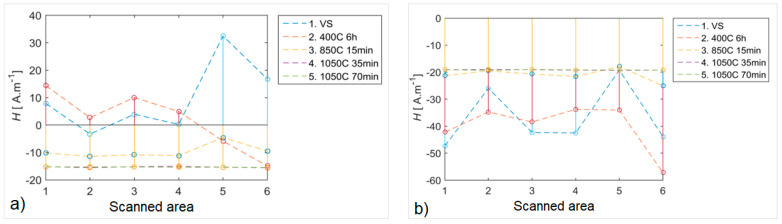
Comparison of (**a**) maximum and (**b**) minimum values on the scanned surfaces of all specimens.

An interesting fact is the comparison of the position of the maxima and minima of the magnetic field of the specimens. Figure 10a,b show the values of the maximum or minimum signal for individual sides of the specimen referred to as scanned area. While the reference specimen VS has the strongest response on sides 5 and 6 (the narrowest block area), specimen No. 2 with a heat treatment duration of *t* = 6 h has the strongest response on the remaining sides. The response of specimen No. 3 is weaker, but the course remains consistent with the reference specimen VS. 

Based on the achieved results of the conducted experiments, it can be stated that the influence of heat treatment in the production of austenitic steels has a non-negligible effect on the mechanical and electromagnetic properties of austenitic steels. The most significant changes include the observed changes in the microstructure of materials, and the disappearance of the presence of some components (δ-ferrite, inclusions, etc.). Furthermore, the effect of heat treatment affects the magnetic field of the investigated structures, while its detectability decreases with the basic factors of this treatment, namely with increasing annealing temperature and the time the specimens are exposed to this temperature. In the conducted experiments, the measurability of the response of the material up to the limit of recrystallization annealing was shown. Above this limit, the material has lost ferromagnetic properties, which is evidenced not only by the measurements in question but also by microstructural images from an electron microscope.

### 3.2. Plastic Deformation Inspection Results

**Figure 11:**
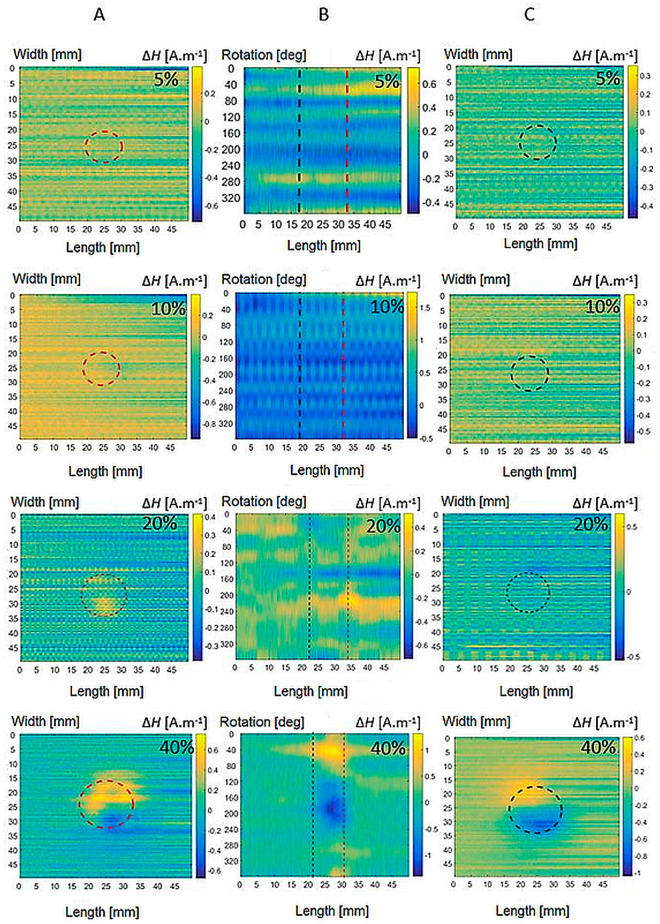
Magnetic field distribution of bases (**A**,**C**) and shell (**B**) of AISI 316L specimens of all plastic deformation.

**Figure 12:**
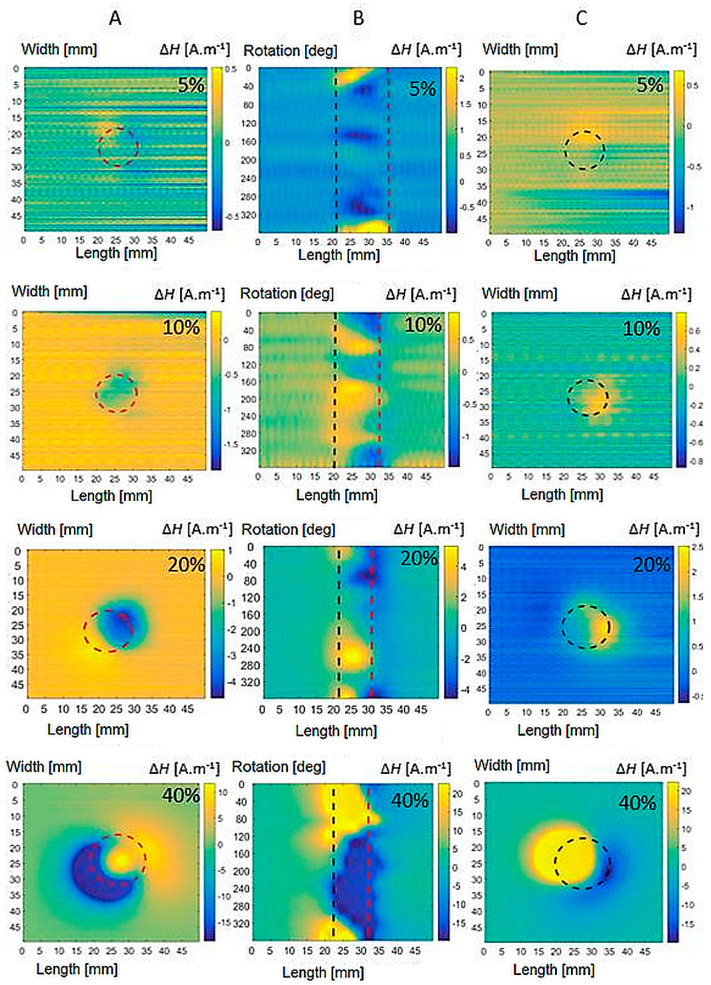
Magnetic field distribution of bases (**A**,**C**) and shell (**B**) of AISI 316Ti specimens of all plastic deformations.

**Figure 13:**
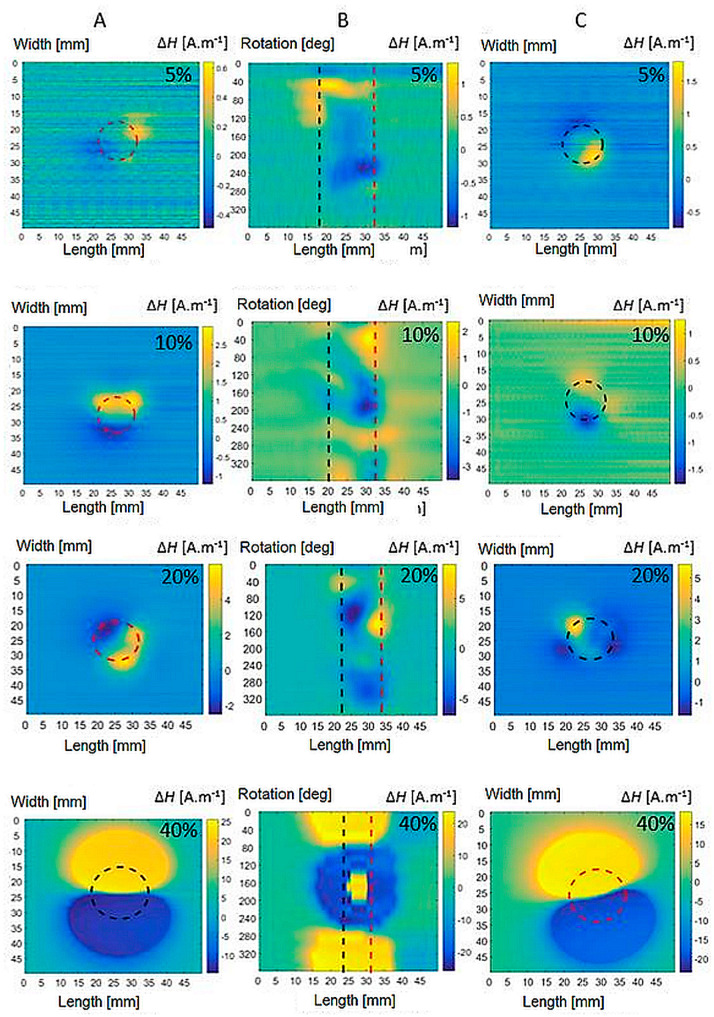
Magnetic field distribution of bases (**A**,**C**) and shell (**B**) of AISI 304 specimens of all plastic deformations.

First, measurements are made on reference specimens without plastic deformation, labelled VS. These signals are measured to be able to further compare and subtract the relevant signals from the other specimens to eliminate the background noise. Furthermore, specimens with the presence of plastic deformation are subjected to measurements. In the next part, it can be seen from the obtained waveforms that individual types of materials show a different magnetic response to the same degree of plastic deformation. This is related to the different representations of individual chemical elements in steels. As the value of the deformation force increases, there is a formation of a greater amount of induced martensite in the biomaterial. This has the effect of strengthening the internal magnetic field of material. The goal of all measurements is the detection of changes in the magnetic field at the greatest possible distance in a non-contact manner. Figure 11 shows the detected magnetic field of the specimens of both the platforms and the shell for AISI 316L of all plastic deformations PD = <5, 40>%. The results show the inhomogeneous nature of the magnetic field. Figure 12 shows that the AISI 316Ti magnetic field values are also well-recorded. The magnetic field of AISI 304 steel, Figure 13, already reaches at 10% deformation values, where the influence of noise can be neglected even without subtraction. The position of the specimen in scans is depicted by its borders drawn as black dashed lines in figures. The degree of plastic deformation is shown in the top right corner of each graph in all pictures. Capital letters A, B, and C on the top of each figure denote the graphs’ column belonging to the specimen’s particular side.

**Figure 14:**
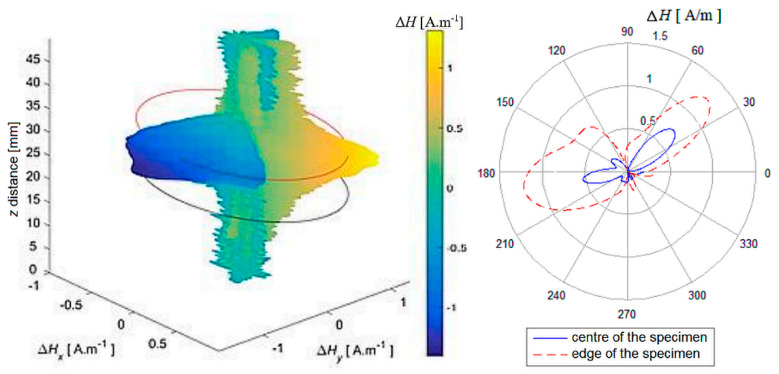
Distribution and reconstruction of the magnetic field of the AISI 316L shell, PD = 40%.

At the degree of plastic deformation PD = 40%, an increase in the field intensity can be observed, while the field is distributed in a larger area around the material. Figure 12, Figure 13 and Figure 14 in the bottom row show a detected magnetic field at PD = 40% for all inspected materials. The field reaches uniform maxima and minima on the bases of the specimen. The achieved results further document that AISI 316L steel exhibits the weakest magnetic field at 40% plastic deformation. AISI 316Ti steel with Ti admixture shows approximately the same manifestations at the same degree of deformation, but the value of the magnetic fields is more pronounced compared to AISI 316L. The strongest generated magnetic field is represented by the results for AISI 304 steel. Even at lower degrees of deformation, it exceeds the field values of the AISI 316 types of steel.

In the next part, three-dimensional reconstructed graphs are presented, which can provide a summary of information about the distribution of the magnetic field on and around the entire examined specimen. The following method was used to create the graphs: individually scanned data (scanning lines of the surface) were grouped in order and plotted as a spatial dependence. The measured value of the intensity of the magnetic field **H** was transferred to the 3D space depending on the rotation angle α of the specimen during scanning according to the Formulas (1) and (2). This preserves the position at which the value was scanned, and the 3D space represents the surface of the specimen, which is represented as a segment with a value of *H* = 0 A.m^−1^ with a length *z* that corresponds to the length of the scanned line of the surface of the specimen shell and the surroundings.

Δ𝐻𝑥 = Δ𝐻. sin 𝛼(1)

Δ𝐻𝑦 = Δ𝐻. cos 𝛼(2)

At the same time, the selected data were displayed using a polar graph for better clarity. It shows the plotted values from the centre of the specimen and from the upper base of the specimen, where the respective values from the sections of the reconstructed field are compared.

**Figure 15:**
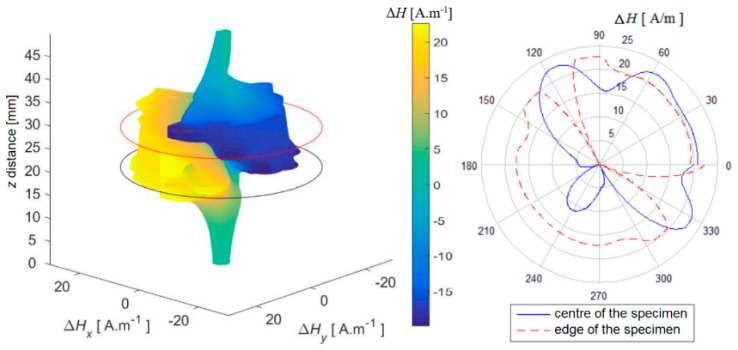
Reconstruction of the magnetic field of the shell. AISI 316Ti, PD = 40%.

**Figure 16:**
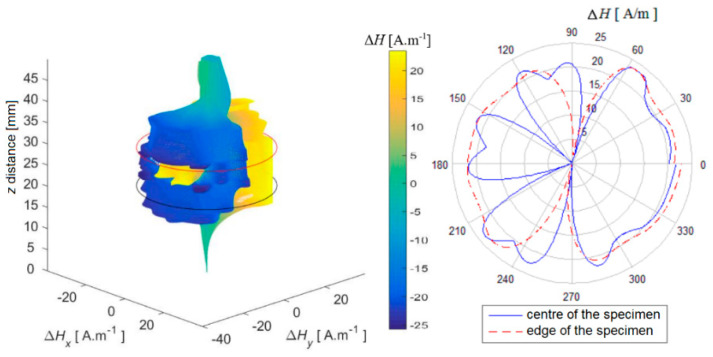
Reconstruction of the magnetic field of the shell. AISI 304, PD = 40%.

In each figure, the contours of the specimen platforms (red and black circles—the beginning and end of the position of the specimen base in the scanned space) and, simultaneously, the colour-coded course of the magnetic field intensity on the specimen in its selected surroundings, are shown in Figure 14, Figure 15 and Figure 16.

It is also possible to notice the unevenness of the signals from the surface of the AISI 304 specimen in this reconstructed image (Figure 16). It can be assumed that the dominant martensitic region is in the centre of mass of the specimen closer to the side with the local minimum, in which the local maximum breaks through the surface.

Based on the above results, the shape of the magnetic field around the specimen varies unevenly and is individual for each specimen, as regards the shape of the field distribution and its intensity.

The results are also presented in the form of graphical dependencies of the voltage response of the fluxgate sensor as a function of the degree of plastic deformation. The procedure for plotting individual functional values is as follows: the maximum value from the examined area is considered, from which the median value of the noise background for the given specimen is subtracted.

**Figure 17:**
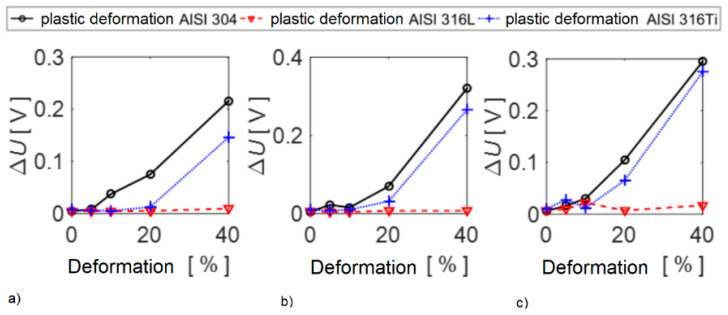
Dependence of the change in the voltage response of the sensor on the degree of deformation—(**a**) top base, (**b**) shell surface, (**c**) bottom base.

The graphs in Figure 17 show the maximum recorded values. Under these conditions, electromagnetic noise from surrounding devices in the premises of the laboratory of electromagnetic methods, where the measurements were performed, did not affect the response. From the dependences shown, the response of the sensor increases with the degree of the deformation rate. The steepest increase in the response can be observed for specimens from the AISI 304 material. However, this increasing trend can be observed for all investigated materials. In the case of AISI 316L steel, this increase is an order of magnitude weaker, and in the case of AISI 316L biomaterial, i.e., low-carbon steel, the observed effect is the weakest. When investigating this biomaterial from greater distances, a situation occurs when the response of the measured quantity using sensors can no longer be captured. 

## 4. Conclusions

This paper was focused on non-destructive evaluation of electrically conductive austenitic biomaterials using a sensitive fluxgate sensor. Three different materials were investigated in terms of various grades of austenitic steels (AISI 304, 316L, and 316Ti). Various heat treatment methods and mechanical deformation levels were applied. The sensed signals were processed and evaluated using the designed algorithm in the LabVIEW environment. For this purpose, specific mathematical operations were used. The goal was to analyse the response of the investigated samples in terms of detecting their intrinsic magnetic field. A quantitative evaluation of such responses was crucial for further use of the sensors and the measurement methods in biomedical practice.

The presented results showed that the influence of heat treatment and mechanical stress, specifically plastic deformation, had a significant effect on the mechanical and electromagnetic properties of the investigated austenitic steels. As the rate of applied plastic deformation increases, microstructural changes occur in the material, which leads to an increase in ferromagnetic components in its structure. As the degree of deformation increases, the value of the magnetic field strength around the specimen increases, which is recorded by a sensitive magnetic field sensor. The sensed and mathematically processed intrinsic magnetic field of the specimens showed its strongly non-homogeneous character. 

The experiments carried out in laboratory conditions showed that, in this way, it is possible to record such macroscopic changes in the properties of biomaterials, which is supported by the achieved results. By using a more sensitive sensor, it would be possible to record these response signals at greater distances, which is the main goal in biomedical practice, given the anatomical conditions when investigating conductive implants in the human body in a non-invasive way.

## Figures and Tables

**Table 1:** Investigated specimens of biomaterial AISI 316L and heat treatment parameters.

Specimen	*T* (°C)	*t* (min)	Vickers Hardness	Description of Treatment
1	0	0	256 HV 0.5	-
2	400	360	256 HV 0.5	suppressing the memory effect of the material
3	850	15	226 HV 0.5	complete recrystallization, reduction of dislocation density in the material
4	1050	35	151 HV 0.5	solution annealing
5	1050	70	150 HV 0.5	solution annealing with the elimination of δ-ferrite

## Data Availability

The data presented in this study are available on request from the corresponding author.

## References

[1] Ratner B.D., Hoffman A.S., Schoen F.J., Lemons J.E. (1996). Biomaterial Science: An Introduction to Materials in Medicine.

[2] Davis J.R., ASM International (2003). Handbook of Materials for Medical Devices.

[3] Yang J., Wang Y., You Z., Liu G., Li B., Zhang G. Study on Micro Fluxgate Magnetic Sensor. European Space Components Information Exchange System Website. https://escies.org/download/webDocumentFile?id=1613.

[4] Areiza M.C.L., Sacremento R., Sommer R.L., González Chávez D.E., Rebello J.M.A., Rao B.P.C., Jayakumar T., Balasubramanian K., Raj B. (2012). Understanding sigma phase influence on the magnetic behaviour of duplex stainless steel. Electromagnetic Nondestructive Evaluation (XV).

[5] Atlas Specialty Metals (2003). The Atlas Specialty Metals Technical Handbook of Stainless Steels.

[6] Davis J.R., Davis J.R. (1994). ASM Specialty Handbook: Stainless Steels.

[7] McGuire M.F. (2008). Austenitic Stainless Steels. Stainless Steels for Design Engineers.

[8] Avrin W.F., Cordell T.M., Rempt R.D. (2000). Eddy current measurements with Magnetoresistive sensors: Third-layer flaw detection in a wing-splice structure 25 mm thick. Nondestructive Evaluation of Aging Aircraft, Airports, and Aerospace Hardware IV, Proceedings of the SPIE 5th Annual International Symposium on Nondestructive Evaluation and Health Monitoring of Aging Infrastructure, Newport Beach, CA, USA, 7–8 March 2000.

[9] Yang G., Tamburrino A., Udpa L., Udpa S.S., Zeng Z., Deng Y., Que P. (2010). Pulsed eddy-current based giant magnetoresistive system for the inspection of aircraft structures. IEEE Trans. Magn..

[10] Rosado L.S., Cardoso F.A., Cardoso S., Ramos P.M., Freitas P.P., Piedade M. (2014). Eddy currents testing probe with magneto-resistive sensors and differential measurement. Sens. Actuators A Phys..

[11] Sukhorukov V.V., Slesarev D.A., Vorontsov A.N. (2014). Electromagnetic Inspection and Diagnostics of Steel Ropes: Technology, Effectiveness and Problems, ET. Mater. Eval..

[12] Liu S., Sun Y., Jiang X., Kang Y. (2020). A Review of Wire Rope Detection Methods, Sensors and Signal Processing Techniques. J. Nondestruct. Eval..

[13] Wang Y., Niu Y., Wei Y., Ye C. (2022). Multi-frequency imaging with non-linear calibration of magnetoresistance sensors for surface and buried defects inspection. NDT E Int..

[14] Tsukada K., Hayashi M., Nakamura Y., Sakai K., Kiwa T. (2018). Small Eddy Current Testing Sensor Probe Using a Tunneling Magnetoresistance Sensor to Detect Cracks in Steel Structures. IEEE Trans. Magn..

[15] Wei S., Liao X., Zhang H., Pang J., Zhou Y. (2021). Recent Progress of Fluxgate Magnetic Sensors: Basic Research and Application. Sensors.

[16] Schoinas S., El Guamra A.-M., Moreillon F., Passeraub P. (2020). Fabrication and Characterization of a Flexible Fluxgate Sensor with Pad-Printed Solenoid Coils. Sensors.

[17] Zhi S., Feng Z., Lei C. (2019). Improved Performance of Fundamental Mode Orthogonal Fluxgate Using a Micro-Patterned Meander-Shaped Ribbon Core. Sensors.

[18] Sun X., Feng Z., Zhi S., Lei C., Zhang D., Zhou Y. (2017). An integrated microfluidic system using a micro-fluxgate and micro spiral coil for magnetic microbeads trapping and detecting. Sci. Rep..

[19] Lei C., Sun X.-C., Liu C., Lei J., Wang T., Yang Z., Zhou Y. (2014). Detection of Dynabeads in small bias magnetic field by a micro fluxgate-based sensing system. J. Appl. Phys..

[20] Smetana M., Čápová K., Chudáčik V., Palček P., Oravcová M. (2016). Plastic Deformation Influence on Intrinsic Magnetic Field of Austenitic Biomaterials. J. Electr. Eng..

[21] Chudacik V., Smetana M., Čápová K., Palček P., Behúň L. Influence of the plastic deformation on intrinsic magnetic field of austenitic biomaterials. Proceedings of the 2017 11th International Conference on Measurement.

[22] Oka M., Yakushiji T., Enokizono M. (2015). Evaluation of the Material Degradation of Austenitic Stainless Steel under Pulsating Tension Stress Using Magnetic Method. J. Jpn. Soc. Appl. Electromagn. Mech..

[23] Elson L., Meraki A., Rushton L.M., Pyragius T., Jensen K. (2022). Detection and Characterisation of Conductive Objects Using Electromagnetic Induction and a Fluxgate Magnetometer. Sensors.

[24] Borgioli F. (2020). From Austenitic Stainless Steel to Expanded Austenite-S Phase: Formation, Characteristics and Properties of an Elusive Metastable Phase. Metals.

[25] Liu S., Long M., Ai S., Zhao Y., Chen D., Feng Y., Duan H., Ma M. (2020). Evolution of Phase Transition and Mechanical Properties of Ultra-High Strength Hot-Stamped Steel During Quenching Process. Metals.

